# Orientation dependent CD45 inhibition with viral and engineered ligands

**DOI:** 10.1126/sciimmunol.adp0707

**Published:** 2024-10-25

**Authors:** Marta T. Borowska, Liu D. Liu, Nathanael A. Caveney, Kevin M. Jude, Won-Ju Kim, Takeya Masubuchi, Enfu Hui, Robbie G. Majzner, K. Christopher Garcia

**Affiliations:** 1 Department of Molecular and Cellular Physiology, Stanford University School of Medicine, Stanford, CA 94063, USA.; 2 Howard Hughes Medical Institute, Stanford University School of Medicine, Stanford, CA 94063, USA.; 3 Department of Pediatric Oncology, Dana-Farber Cancer Institute, Harvard Medical School, Boston, MA 02115, USA.; 4 Section of Cell and Developmental Biology, Division of Biological Sciences, University of California, San Diego, La Jolla, CA 92093, USA.; 5 Department of Structural Biology, Stanford University, Stanford, CA 94063, USA.

## Abstract

CD45 is a cell surface phosphatase that shapes the T cell receptor signaling threshold but does not have a known ligand. A family of adenovirus proteins, including E3/49K, exploit CD45 to evade immunity by binding to the extracellular domain of CD45, suppressing T cell signaling. We determined the cryo-EM structure of this complex and found that the E3/49K protein is composed of three Ig-domains assembled as “beads on a string” and forces CD45 into a closely abutted dimer by cross-linking the CD45 D3 domain, leading to suppression of its intracellular phosphatase activity. Inspired by the E3/49K mechanism, we engineered CD45 surrogate ligands that can fine-tune T cell activation by dimerizing CD45 in different orientations and proximities. The adenovirus E3/49K protein has taught us that, despite a lack of a known ligand, CD45 activity can be modulated by extracellular dimerizing ligands that perturb its phosphatase activity and alter T cell responses.

## One Sentence Summary

We describe a structure of adenoviral E3/49K complexed with CD45, which provides a mechanistic blueprint for engineering T cell immunomodulators.

## INTRODUCTION

Fine-tuned regulation of T cell signaling determines T cell immune tolerance and proliferation, and its perturbation can lead to autoimmunity or anergy (1). CD45 is a cell surface phosphatase necessary for T cells to effectively initiate and propagate immune responses (2–5). Its broad expression on hematopoietic cells makes it an indispensable rheostat for adaptive and innate immune responses. The primary role of CD45 lies in regulating signal transduction events within immune cells by controlling the phosphorylation status of tyrosine residues on various signaling proteins, such as lymphocyte-specific kinase (Lck) (6–8). Lck initiates the T cell receptor (TCR) signaling cascade inside the cell by phosphorylating Zeta-associated protein 70 (Zap70) and immunoreceptor tyrosine-based motifs within the TCR-associated chains (9). Whereas CD45 contains a large extracellular domain (ECD), it has no known endogenous ligand. Indeed, excluding CD45 from the immunological synapse via its large ECD prolongs TCR signaling within the immunological synapse by segregating its intracellular phosphatase activity away from its substrates (6, 10). Therefore, CD45 is necessary for T cell functions mediated via TCR signaling.

This critical role of CD45 has been exploited by viruses to evade immunity. CD45 is targeted by mouse cytomegalovirus (mCMV) and human herpes virus (hHCV), which have been reported to downregulate the expression of CD45 using intracellular mechanisms (11). By contrast, human CMV protein pUL11 and adenoviral strain D protein E3/49K, are expressed as membrane tethers and are proposed to act extracellularly to dimerize CD45 and downregulate its phosphatase activity (12, 13). As previously published, anti-CD45 crosslinking strategies lead to reduced phosphatase activity (14–16).

This viral exploitation of CD45, a cell surface protein with no known ligand, to alter T cell signaling in vivo provides a natural proof-of-concept that CD45 activity can be modulated by ligands that bind to its extracellular domain, which could have biotechnology and therapeutic applications. Previously, monoclonal Abs to CD45 have been tested for differential activation of T cells and as lymphodepletion agents, specifically hematopoietic cell depletion in patients receiving bone marrow transplants (14, 17). However, broad-based depletion of CD45 expressing cells, given its very broad distribution and very high expression on T, B, and myeloid cells, would likely only be helpful for short-term preconditioning. Therefore, a mechanism-based, tunable modulation of the CD45 phosphatase activity that spares responder cells, as affected by viruses, might have clinical utility as a strategy for autoimmune and inflammatory diseases.

Here, we determine the atomic structure of viral E3/49K engaging CD45 and elucidate its orientation-dependent molecular mechanism of CD45 activity modulation. Further, we explore the potential of targeting CD45 for immunomodulation through structure-based engineering of viral E3/49K and synthetic mimics. We show an array of fine-tuned, orientation-dependent binders that either enhance or suppress T cell activation. Our results show a promising new way to modulate T cell activation.

## RESULTS

### Adenoviral E3/49K strain D19a shows binding to CD45 and inhibits T cell proliferation

**Fig. 1.**
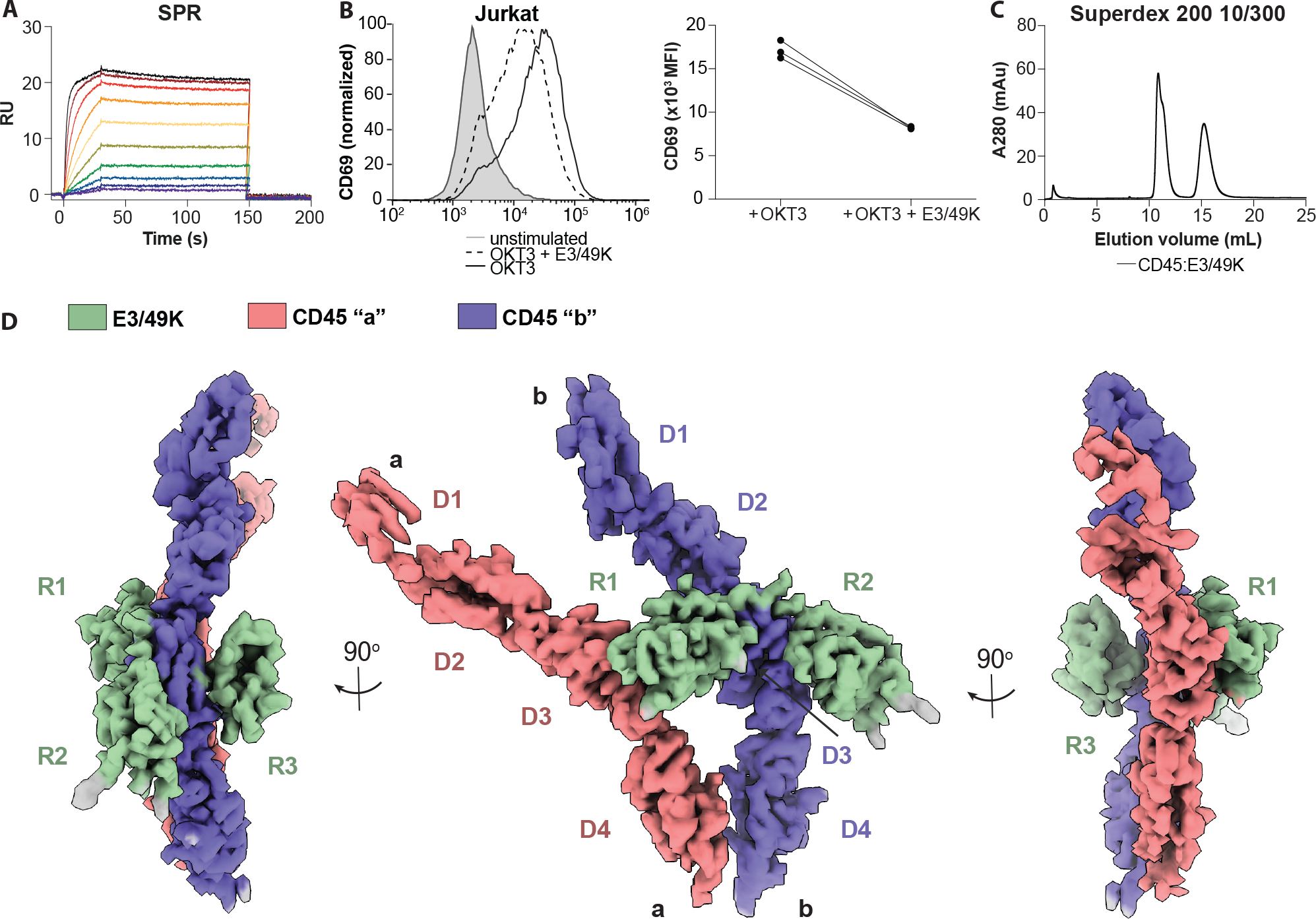
Biochemical characterization and cryoEM structure of CD45:E3/49K complex. **(A)** SPR plot of CD45 and E3/49K strain D19a. **(B)** Example CD69 expression upon pretreatment with E3/49K then stimulated with OKT3 in Jurkat T cells (left) and quantification from replicates (right). **(C)** Superdex 200 size-exclusion chromatography CD45:E3/49K complex. **(D)** CryoEM density map of CD45 dimer (red and purple) bound by a single E3/49K (green) resolved to 3.8-Å resolution.

To assess the binding between human full-length CD45 extracellular domain (ECD) isoform RABC and adenoviral D19a strain E3/49K ECD, we purified both recombinant proteins individually and then measured their affinity to be 0.3 nM (k_on_ = 2.084 *10^6^ 1/Ms, k_off_ = 5.978 *10^−4^ 1/s) by surface plasmon resonance (SPR) (Fig. 1A). Additionally, staining of both Jurkat WT cell and J45.01, a CD45 knockout Jurkat cells, with E3/49K showed that CD45 is the primary binding target for E3/49K (fig. S1A). We validated that our recombinant E3/49K ECD is functional by incubating it with anti-CD3 antibody treated Jurkat cells and detecting the T cell activation marker CD69, as previously shown (12). We observed a considerable inhibition of CD69 upregulation in the presence of soluble E3/49K, in agreement with previously published data (Fig. 1B) (18). To further determine the functional concentration range of E3/49K protein in Jurkat cells, we titrated E3/49K protein in our assay, which resulted in a “U” shape correlation between E3/49K concentration and CD69 expression. This suggests E3/49K might have more than one binding site and higher concentrations of E3/49K shift the complex to a 1:1 stoichiometry (fig. S1B).

To gain insight into the structure of this complex, we purified fully glycosylated human CD45 ECD bound to E3/49K by size exclusion chromatography and prepared cryo-EM grids (Fig. 1C, fig. S1C). Three-dimensional reconstruction of selected particles generated a 3.8-Å nominal resolution map of 2:1 CD45:E3/49K complex (Fig. 1D, and fig. S1, D to E, and fig. S2A, and table S1). Structure docking of both CD45 ECDs matched a previously published crystal structure (PDB ID: 5FMV), and E3/49K domains are predicted to form three Ig-folds by AlphaFold2 (19, 20). We observed density for all three R1-R3 domains of E3/49K binding to CD45, despite R3 having no detectable affinity to CD45 by SPR compared with 74 and 145 nM affinities by R1 and R2, respectively (fig. S1F).

### Viral E3/49K engages two CD45 molecules

**Fig. 2.**
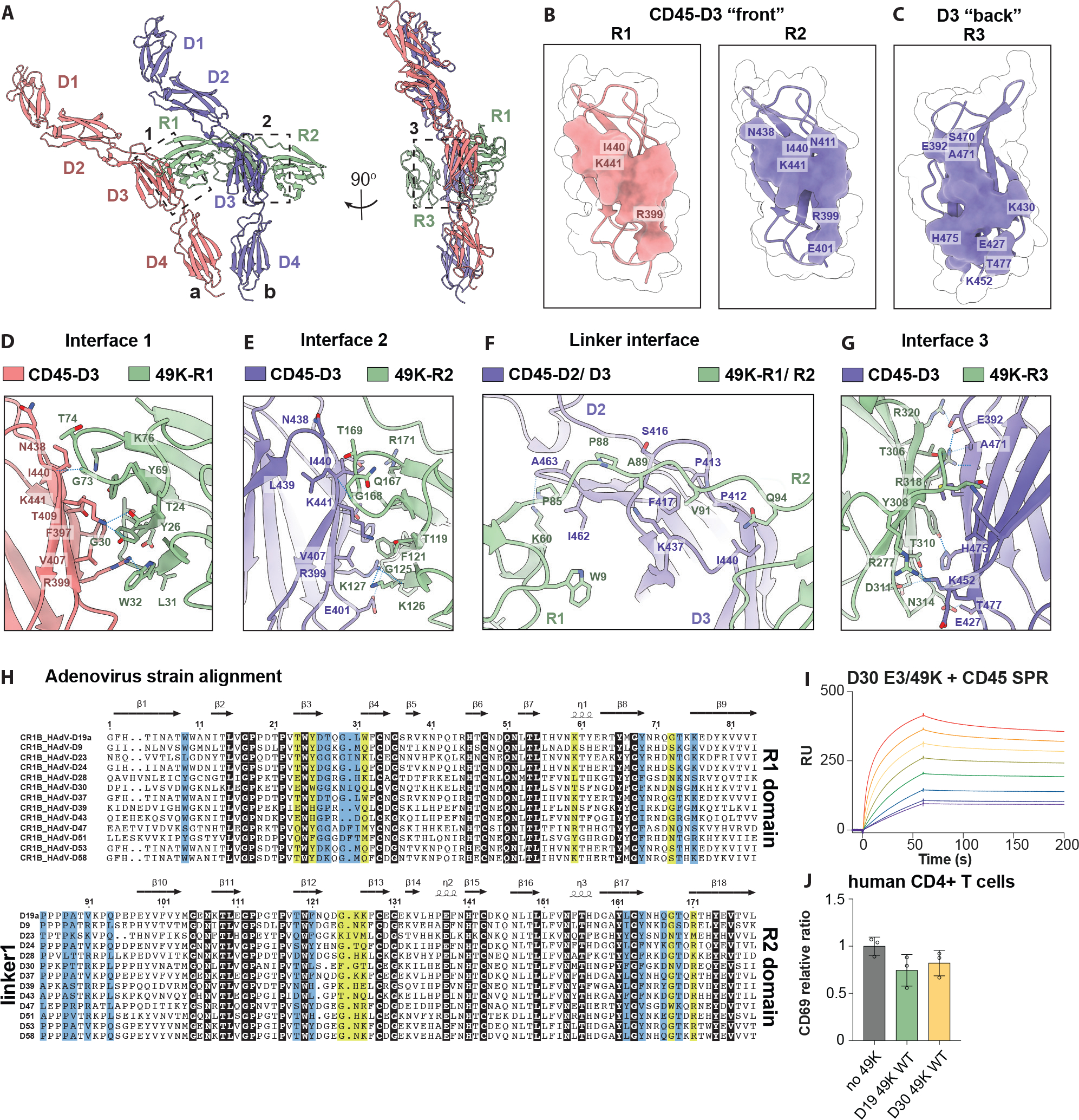
Structural basis for CD45 engagement by viral E3/49K. **(A)** Ribbon diagram of the 2:1 CD45:E3/49K complex. Dashed boxes indicate magnified interface views in later panels. **(B)** Binding footprint of E3/49K R1 (left) and -R2 (right) to the front of CD45 D3. **(C)** Binding footprint of E3/49K-R3 to the back of CD45-D3. **(D)** Interface 1 view of CD45-D3 and E3/49K R1. **(E)** Interface 2 view of CD45-D3 and E3/49K R2. **(F)** Interface view of CD45-D3 and linker between E3/49K R1-R2. **(G)** Interface 3 view of CD45-D3 and E3/49K R3. **(H)** Amino acid sequence alignment of E3/49K R1, linker and R2 between different D strains of adenovirus using Jalview (2.11.0). Highlighted residues are conserved (black), H-bond contacts (yellow) or vdW contacts (light blue). Arrows and helices indicate secondary structures. **(I)** SPR plot of CD45 and E3/49K strain D30. **(J)** CD69 expression upon incubating with E3/49K strain D30 compared with D19a, then stimulating with OKT3.

In the complex, CD45 is an asymmetric dimer that are positioned in *cis* and shifted at approximately 30-degree angle relative to one another. All CD45 domains are resolved, except the most external region of the D1 domain, conveying flexibility from the large N-terminal mucin domain unaligned in this volume map (fig. S1E). The membrane-proximal regions of two CD45 molecules are in close apposition, likely translating that proximity to its intracellular domains such that the phosphatase domains would be sterically hindered from free access to their substrates. E3/49K uses a “beads on a string” interaction mode with CD45: all three domains of E3/49K protein converge on the CD45 D3 domain, but each uses a different contact surface, resulting in an asymmetric complex. The CD45 D3 domain contains three entirely distinct interaction interfaces with different domains of E3/49K. The E3/49K R1 and R2 domains engage the “front” and R3 with the “back” face of D3 domains in a perpendicular and parallel manner, respectively (Fig. 2A to C). The R1 and R2 domains share an overlapping, but molecularly distinct, docking site at the “front” face of CD45 D3 domain with ~ 682-Å and ~ 430-Å of non-polar and ~ 596-Å and ~ 436-Å polar buried surface areas, respectively (table S2). However, despite both interfaces centering around residues Arg^399^ and Ile^440^ of CD45 D3, they use starkly different detailed modes of engagement (Fig. 2B, and table S3). The R1–D3 domain interface of CD45 monomer “a” is mediated by four hydrogen bonds (H-bonds), and many van der Waals (vdW) interactions involving R1 residues Tyr^26^, Thr^28^, Gly^30^, Leu^31^, Trp^32^ contacting mostly Arg^399^ and Thr^24^, Tyr^69^, Gly^73^, Thr^74^ and Lys^76^, which contact around Ile^440^ (Fig. 2D, and table S3). Strikingly, Lys^60^ of R1 also forms additional H-bond with the other CD45 D3 domain. The R2–D3 domain of CD45 monomer “b” is also focused around two cores with six H-bonds and flanked by tightly packed vdW contacts (Fig. 2E, and table S3). The Arg^399^ core includes residues Phe^121^, Gly^125^, Lys^126^, Lys^127^ and Leu^162^, whereas the Ile^440^ core contacts Thr^119^, Tyr^164^, Gln^167^, Gly^168^, Thr^169^, Arg^171^ (Fig. 2E, and table S3). Interestingly, the short, proline-rich linker between R1 and R2 domains also forms vast contacts with CD45 monomer “b” and together with Lys^60^ from R1 domain, can possibly orchestrate a specific relative orientation of two CD45 molecules (Fig. 2F, and table S3). R3 domain maps to the “back” face of D3 of CD45 monomer “b”, with striking nine H-bonds and three salt bridges, covering ~ 742-Å of non-polar and ~ 576-Å of polar buried surface area, despite no detectable affinity by SPR (Fig. 2, C and G, and fig. S1G, and tables S2 to S3).

A previously published protein-protein interaction screen showed all E3/49k-like proteins from adenoviral strain D bind to CD45; however, their amino acid sequences are not highly conserved (Fig. 2H) (21). To see whether E3/49K homologs from diverse D strains still bind to CD45, we tested E3/49k-like proteins from strain D30 as an example and observed comparable binding affinity and T cell signaling inhibition, as well as low-resolution 2D classification of D30 E3/49K:CD45 complex implies similar binding mode (Fig. 2, I to J, and fig. S1H). This suggests E3/49K binding to CD45 is common across many adenoviral D strains.

### R1R2 domains of E3/49K and its VHH dimeric mimics are sufficient to downregulate T cells

**Fig. 3.**
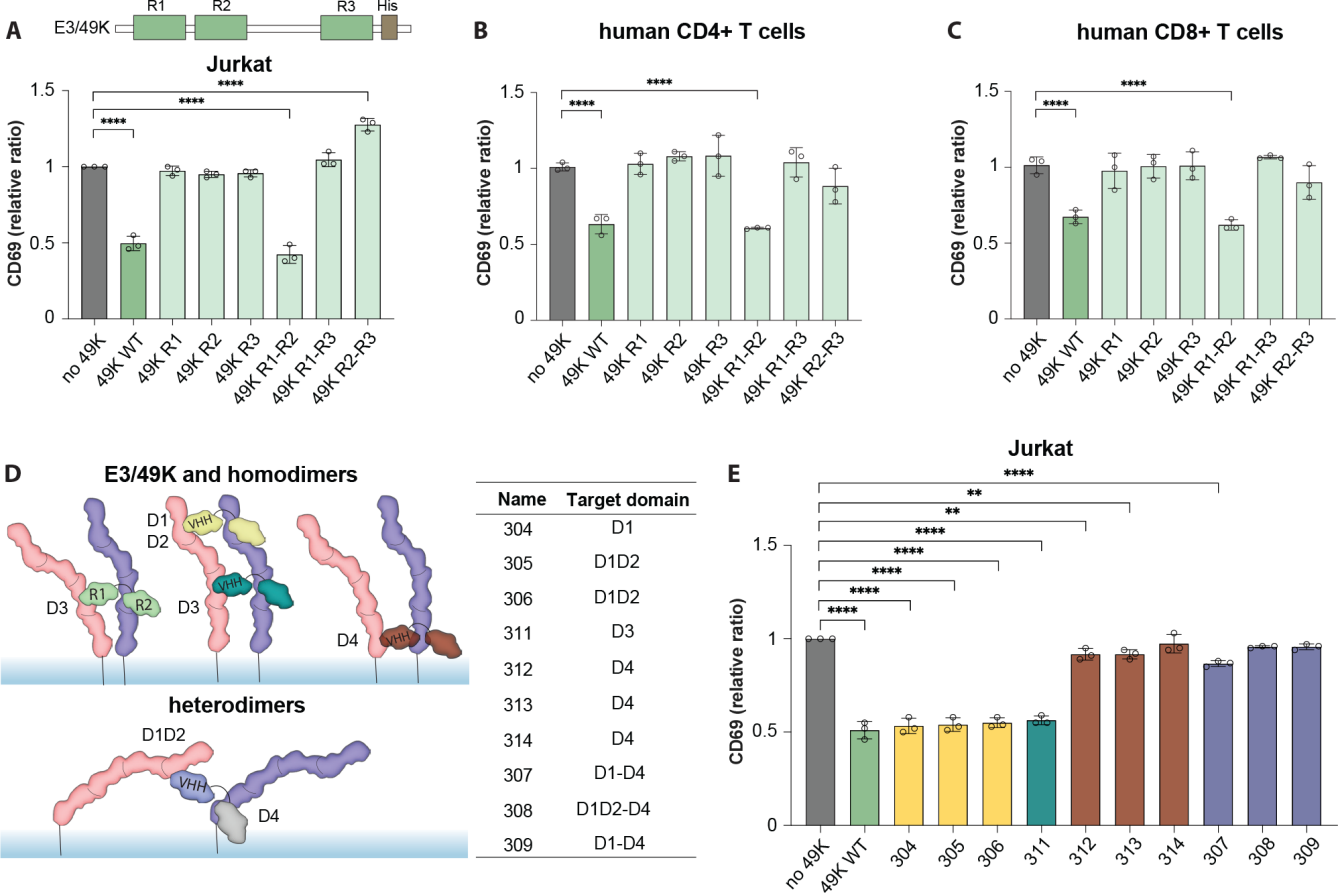
R1R2 domains of E3/49K and its VHH mimics are sufficient to downregulate T cells. **(A)** (*top*) Cartoon representation of E3/49K construct and its R1-R3 domains. (*bottom*) CD69 expression upon incubating with different combinations of linked E3/49K domains, then stimulating with OKT3. **(B)** Same constructs tested in PBMC CD4+ T cells and **(C)** CD8+ T cells. **(A-C)**, Data are mean ± s.d. from n = 3 replicates from different donors. Statistical significance is determined by one-way ANOVA with Fisher’s LSD multiple comparison test, (ns > 0.05; *P < 0.05; **P < 0.01). **(D)** (left) Cartoon representation of R1-R2 dimerizer and its VHH mimics binding to different domains of CD45 and inducing different conformations. (*right*) Table detailing targeted CD45 domains by each tested VHH mimic. **(E)** CD69 expression upon pretreatment with different VHH mimics then stimulated with OKT3 in Jurkat T cells. Data are mean ± s.d. from n = 3 replicates. Statistical significance is determined by one-way ANOVA with Fisher’s LSD multiple comparison test (ns > 0.05; *P < 0.05; **P < 0.01, ***P < 0.001; ****P < 0.0001).

It was previously reported that R1R2-induced CD45 dimerization is sufficient to inhibit CD45 and dampen T cell activation (18). To confirm this, we generated a panel of combinations of linked E3/49K domains. Indeed, after activation of Jurkat cells with OKT3 and in the presence of R1R2 dimer, T cells show comparable inhibition of CD69 upregulation to full-length E3/49K (Fig. 3A). Neither R1R3 nor R2R3 had any effect on CD69 levels, suggesting that these constructs are unable to dimerize CD45 functionally. This also holds for CD69, CD25 and CD107 upregulation in both human CD4+ and CD8+ primary T cells, and to a lesser extent on PD-1 and Ki67 markers with slower activation kinetics (Fig. 3, B and C, and fig. S3, A–D), suggesting that the general inhibition effect of viral-derived E3/49K depends on its R1R2 domain dimerization of CD45.

While E3/49K proteins have powerful functional activities in vivo, they are unlikely drug candidates for immunosuppression given the prospects for immunogenicity. We asked whether CD45 dimerization could be functionally recapitulated with surrogate molecules as a general ‘tunable’ inhibition strategy for T cell activation. Given the structural similarity of the E3/49K protein to tandem VHH domains, we used previously published anti-CD45 VHH binders that target CD45 domains D1-D4 with high specificity and affinity (22). We then generated VHH homo- and heterodimers by fusing two binders with a two amino acid flexible linker, allowing for the dimerization of CD45 through specific domains (Fig. 3D). In total, ten dimeric binders were tested, where four of them show comparable CD69 inhibition as full-length E3/49K protein in Jurkat cells and two in peripheral blood mononuclear cells (PBMCs) (Fig. 3E, and fig. S4, A–E). Interestingly, these functional binders target the D1, D2, or D3 domains of CD45 protein but not the membrane proximal D4 domain. This suggests that dimerization alone is insufficient to inhibit T cell activity significantly, and that not any dimer will do: the specific relative orientations of two CD45 molecules are required for optimal dimerization.

### CD45 dimerization reduces T cell signaling through Lck and Zap70

**Fig. 4.**
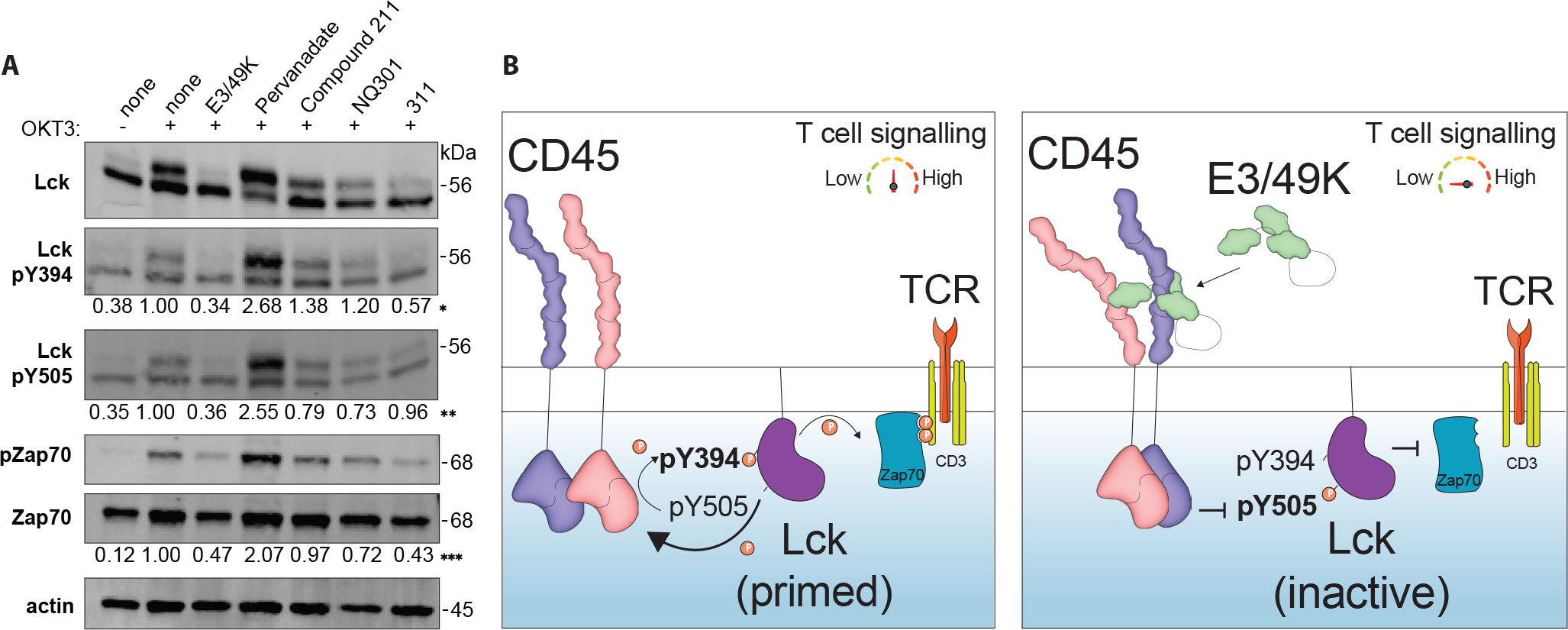
CD45 dimerization reduces T cell signaling through Lck and Zap70. **(A)** Detection of specific phosphorylation in Lck and Zap70 in Jurkat cells by protein immunoblot after various treatments. Compound 211 and NQ301 are purchased from different sources with identical chemical formula. Numbers below panels denote quantification of band intensity normalized as: (*) Lck pY394/Lck ratio, (**) Lck pY505/Lck ratio, (***) pZap70/Zap70 ratio. **(B)** Schematic depiction of CD45-mediated T cell signaling before (left) and after (right) binding of E3/49K binding.

During T cell activation, CD45 initiates a sequential phosphorylation cascade by dephosphorylating Tyr^505^ of the Src family kinase Lck, which releases an intramolecular lock that holds Lck in a closed - inactive state (23). This event leads to intramolecular trans-autophosphorylation of Tyr^394^ and activation of Lck, which then initiates T cell signaling through Zap70 phosphorylation that transduces the signal onto the TCR-CD3 complex (24). E3/49K was previously shown to have an effect on levels of Lck and Zap70 phosphorylation (12), therefore, to understand this mechanism we compared the response of E3/49K to anti-CD45 VHH homodimer (311) and various CD45 phosphatase inhibitors for the CD45 downstream signaling response in Jurkat cells. We chose CD45 specific inhibitors: Compound 211 and NQ301 are identical molecules from different sources, as well as a more promiscuous PTP inhibitor, Pervanadate, which shows dose-dependent inhibition of CD45 (fig. S5, A and B) (25–28). Jurkat cells were pretreated with target molecules for 30 minutes, then stimulated with OKT3 for 5 minutes, and further assessed by protein immunoblot. Consistent with defective T cell activation signaling in these cells, both E3/49K and 311 treatment leads to decreased phosphorylation at both Lck Tyr^394^ and Tyr^505^, as well as downstream Zap70 (Fig. 4A, fig. S5, C–F). However, Compound 211 and NQ301 inhibitors showed no change in phosphorylation, in contrast, Pervanadate treatment led to a pronounced increase in phosphorylation of all sites (Fig. 4A). These experiments suggest that E3/49K effectively inhibits CD45 and suppresses downstream T cell signaling through a direct effect on its ability to activate Lck and Zap70, compared to the more pleiotropic chemical inhibitors (Fig. 4B).

### CD45 distancing tunes T cell signaling

**Fig. 5.**
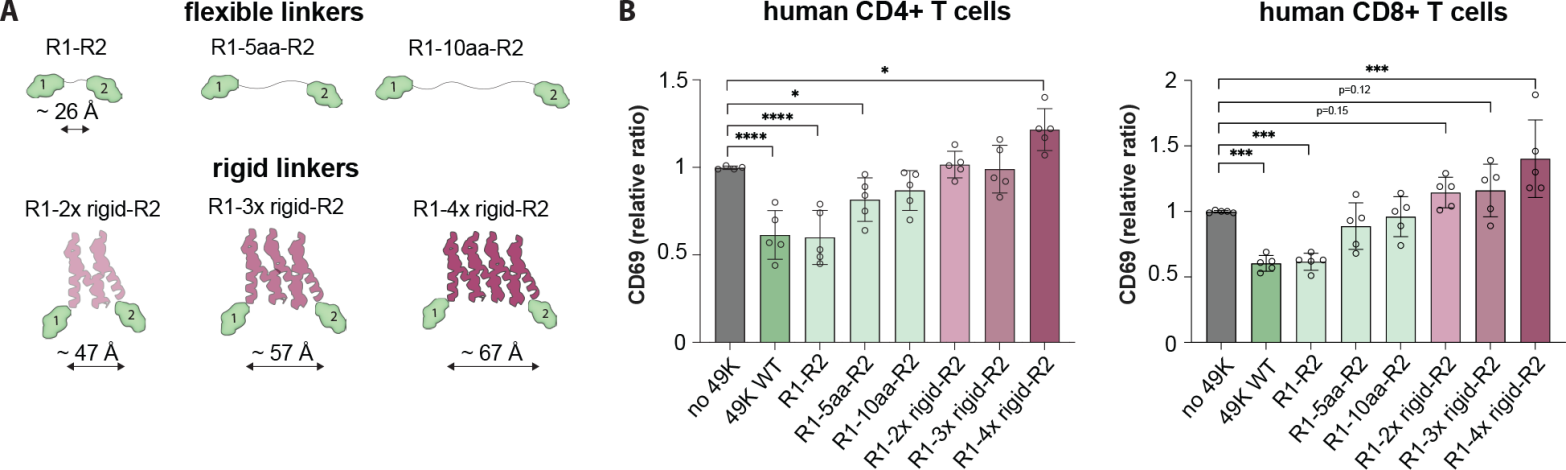
CD45 distancing tunes T cell signaling. **(A)** Cartoon representation of E3/49K constructs with extended flexible or rigid linkers. **(B)** PBMCs were pretreated with E3/49K with different types of linkers then stimulated with OKT3 and assayed for CD69 expression in CD4+ (*left*) and CD8+ (*right*) T cells. Data are mean ± s.d. from n = 5 replicates from different donors. Statistical significance is determined by a one-way ANOVA with Fisher’s LSD multiple comparison test (ns > 0.05; *P < 0.05; **P < 0.01, ***P < 0.001; ****P < 0.0001).

We also engineered E3/49K variants to alter the topological distance between CD45 molecules by introducing flexible or rigid linkers between the CD45 binding domains. The flexible linkers consisted of 5 to 10 amino acid-long Gly-Ser inserts between R1 and linker-R2 domains, while rigid linkers are composed of planar and stackable ankyrin inserts that that would form a rigid ‘wedge’ between R1-R2 binders (Fig. 5A) (29). We observed that all constructs that reverted the effect of E3/49K downregulation correlated with the length and type of the linker in PBMCs, and most constructs in Jurkat cells (Fig. 5B, fig. S6, A–E). Interestingly, the most extended rigid linker, R1–4x insert-R2, further enhances the OKT3-stimulated T cell activation level in PBMCs, possibly due to the enforced separation of phosphatase domains to enhance their activity. Additionally, engineered molecules with 3x and 4x-insert rigid linkers rescue the suppression of CD69 upregulation under Staphylococcal Enterotoxin B (SEB) superantigen stimulation, while no inhibition was observed under cytomegalovirus (CMV) lysate stimulation due to the low ratio of responsive cells among PBMCs (fig. S6, F and G). Our results demonstrate that both orientation and proximity can be used to tune T cell signaling via CD45.

## DISCUSSION

Our study has elucidated the structural mechanism for exploiting human CD45 by the adenoviral D19a strain E3/49K to evade immunity. The CD45-E3/49K complex is a 2:1 asymmetric dimer, where two rigid CD45 molecules are positioned in a *cis* back-to-back orientation and shifted at an approximately 30-degree angle relative to each other. The vicinity of the membrane-proximal regions of the two CD45 molecules implies an influence on their intracellular domains, likely the enforcement of closeness that would sterically impede access of the CD45 phosphatase domains to its substrates such as Lck and Zap70. Inhibition of phosphatase domain by ligand-induced dimerization has been demonstrated before by swapping CD45 ECD with EGFR ECD domains (30). Additionally, it has been reported that alternative spliced domains of CD45, i.e. tall RABC isoform present in Jurkat cells and short R0 isoform present mostly in activated primary cells might modulate that dimerization (31). This is apparent in comparing our Jurkat and PBMCs results, which show lesser effect in PBMCs. One possible mechanism for observed T cell downregulation in the presence of E3/49K is the “inhibitory wedge hypothesis”, which proposes that in the dimer of a wild-type CD45 phosphatase, the catalytic site of one molecule would be blocked by “the wedge”, Glu624 from another molecule, inhibiting activity in both phosphatases and leading to inactive Lck and inhibited T cell signaling (32–33). We do not have evidence for that E3/49K works in this fashion.

Targeting CD45 phosphatase activity is a therapeutic strategy for treating autoimmune diseases, cancer, and organ transplantation (17, 34–38). For example, CD45 is overexpressed in leukemia and lymphoma, and a mutation of CD45 has been shown to reduce CAR T cell recognition yet preserve CD45 function, avoiding on-target/off-tumor toxicities (37). However, no strategies exist to directly modulate CD45 phosphatase activity using extracellular binders that work through induced proximity.

We extended our exploitation of the functional implications of E3/49K by focusing on developing tools to tune T cell activity. Building upon the significance of the linker and induced orientation, we developed a strategy to tune T cell signaling by physically separating two CD45 molecules with binders with different types of linkers. Specifically, by inserting longer flexible or rigid linkers to increase the distance between two R1-R2 binders, we showed we can upregulate T cell activity beyond OKT3 only stimulation level. This approach holds promise for further fine-tuning T cell activity.

While the precise mechanism by which extracellular CD45 inhibition downregulates T cells requires further investigation, the tools to modulate T cell activity we developed in this study show significant therapeutic potential. Additional in vivo studies are needed to evaluate their effectiveness.

## MATERIALS AND METHODS

### Study design

The objective of this study was to describe a structural mechanism by which a family of adenovirus proteins, specifically how E3/49K suppresses T cells via CD45. We used cryo-EM to determine the 3.8-Å structure of the E3/49K-CD45 complex. We elucidate the molecular details of how E3/49K utilizes its Ig-like domains to compel the closure of a CD45 dimer, resulting in the suppression of its intracellular phosphatase activity. Lastly, inspired by the similarity of the E3/49K proteins to tandem nanobodies, we have engineered CD45 surrogate ligands capable of mimicking and further fine-tuning T cell activation. A group of engineered proteins, including the E3/49K subdomain, R1-R2 variants with a series of linker lengths and anti-CD45 nanobody dimers were utilized in the study. In vitro stimulation assays were performed in both Jurkat cells and human primary T cells, with cell markers detected to evaluate the T cell efficacy, proliferation, and exhaustion status. The number (N) of biological replicates is indicated in figure legends. protein immunoblot was conducted to measure the phosphorylation status of downstream signaling. These ligands leverage orientation- and proximity-dependent extracellular dimerization to modulate CD45 activity.

### Cell lines

All cell lines were purchased from ATCC and kept in a humidified incubator at 37 °C with 5% CO_2_. WT and CD45 knocked down Jurkat T cells (J45.01), and primary human T cells were cultured in RPMI (Corning) and supplemented with 10% FBS (Sigma). All cell lines tested negative for mycoplasma (MycoAlert Mycoplasma Detection kit, Lonza).

### Jurkat stimulation

50 μL of Jurkat T cells wild-type and J45.01 were resuspended in fresh medium and seeded at 10^5^ cells/well in a 96-well plate. 50 μL of 100 nM of E3/49K or VHH variants (25 nM final) were diluted in media and added to appropriate wells for 30 min incubation at 37 °C. Then 100 μL of OKT3 (Biolegend) was added at 2 μg/ml (final concentration of 1 μg/ml) into each well and incubated overnight for 12–16 h at 37 °C. Cell activation was determined by FACS using an anti-CD69-FITC antibody (Biolegend).

### PBMC stimulation with OKT3

PBMCs were obtained from the Stanford Blood Bank. Cells in deidentified leukoreduction chambers from healthy platelet donors were processed as soon as possible and stored in LN_2_. Frozen PBMCs were thawed and stimulated with CD3/CD28 Human T-Activator Dynabeads at a bead-to-cell ratio 1:1 and 30 U/ml rIL-2 for 4 days. Then, the expanded cells were rested for another 4 days for the following experiment. 50 μL of expanded T were resuspended in fresh medium and seeded at 10^5^ cells/well in a 96-well plate. 60 nM of E3/49K or VHH variants were used to pretreat cells for 30 min incubation at 37 °C. Then 100 μL of OKT3 (Biolegend) was added at 2 μg/ml (final concentration of 1 μg/ml) into each well and incubated overnight for 6–12 h at 37 °C. Cell activation was determined by FACS using an anti-CD69-FITC antibody (Biolegend).

### PBMC stimulation with SEB and CMV lysates

Frozen PBMCs were thawed and recovered overnight. 50 μL of PBMCs were resuspended in fresh medium and seeded at 10^5^ cells/well in a 96-well plate. 60 nM of E3/49K or variants were used to pretreat cells for 30 min incubation at 37 °C. After the pretreatment, SEB or CMV stimulation was performed. For SEB stimulation, 2.5 μg/mL of S. aureus Enterotoxin B (Sigma-Aldrich) was added and treated together with 1 μg/mL costimulatory antibodies anti-CD28 and anti-CD49d in PBMCs and incubated overnight at 37 °C. For CMV stimulation, CMV lysates (10 μg/mL, Enzo Life Sci) along with a mixture of CMV peptide pools including pp65 and IE-1 protein (1μg/ml each; PepTivator-CMV pp65 and IE-1; Miltenyi Biotec) were used to treat PBMCs. Additionally, 1 μg/mL costimulatory antibodies anti-CD28 and anti-CD49d were added into each well and incubated overnight at 37 °C.

### FACS staining

Cells were stained with the indicated antibodies for 15 min at RT in the dark in MACS staining buffer (Miltenyi). After incubation with human Fc-blocking reagent where appropriate (TruStain, BioLegend) and fluorescent antibodies, cells were washed and analyzed via flow cytometry on CytoFLEX S (Beckman Coulter) instrument. For the surface or intracellular detection of CD3, CD4, CD8, CD25, CD45RO, CD45RABC, CD69, CD107, PD-1, Ki67 markers cells were incubated with appropriate antibodies at 1:200 dilution in MACS buffer. The list of antibodies used in this study is included in table S4. Surface expression was quantified by FACS using the CytoFLEX S, which is equipped with a high-throughput sampler. Live cells were identified after gating based on forward scatter (FSC), side scatter (SSC), and propidium iodide (PI)-negative staining. Gating strategy is shown in fig. S7. Data were analyzed using FlowJo (10.8.1, Tree Star) using geometric mean fluorescence intensity (MFI). All assays were performed using independent biological replicates.

### Protein immunoblotting

Wild-type Jurkat cells were starved overnight in serum-free medium. Next day, 2.5× 10^6^ Jurkat cells were preincubated with tested reagent for 30 min in following concentration, including E3/49K (25 nM), VHH 311 (25 nM), compound 211 (1 uM), pervanadate (100 uM). Subsequently, cells were stimulated with OKT3 at 3 ug/ml final concentration for 5 min. Then, cells were added into 10 ml cold PBS buffer and lysed with RIPA buffer supplemented with protease inhibitors (Protease Inhibitor Cocktail Tablets, Roche). Proteins were separated by SDS-PAGE gel electrophoresis and transferred to PVDF membranes. Membranes were blotted with anti-Lck, anti-LCK pY505, anti-phospho-Src family (Tyr416), anti-Zap70, anti-phospho-zap70 (Tyr319)/Syk (Tyr352), anti-b-Actin antibodies. Blots were incubated with fluor-conjugated secondary antibodies and imaged with LI-COR Biosciences Odyssey imaging system. Quantification was performed with ImageJ software.

### Protein construct designs and expression

Wild-type protein sequences were obtained from Uniprot (https://www.uniprot.org/), and anti-CD45 VHHs sequences were gifted from Synthekine (22). All constructs and its amino acid sequences used in this study are listed in table S5. E3/49K ECD (G1-Q334) and various truncations were cloned into pD649 with either C-terminal Avitag-6x His-tag (for SPR) or 6x His-tag (all other assays). To mimic E3/49K dimerization, two anti-CD45 VHHs were linked with various linkers in a homodimeric or heterodimeric fashion. For flexible linkers 2, 5, or 10 amino acid long linker (GG or GGGGS_n_) were used appropriately. As a rigid linker, we used DHR10 as a scaffold (39), ranging from 1–8 double helical 46 amino acids long repeats. For CD45 ECD (Q1-K552), we used previously reported constructs cloned into pD649 as for E3/49K (12). All proteins were expressed in Expi293 cells using the manufacturer’s protocol. After 4 days, supernatant was harvested and incubated with Ni-NTA resin (QIAGEN) for overnight 4 °C. Ni-NTA beads were collected and washed by gravity column (Bio-Rad) with 20 cv 1× HBS pH 7.2 with 20 mM imidazole and eluted with 5cv 1× HBS pH 7.2 with 250 mM imidazole. Proteins were concentrated using a 30 kDa filter (Millipore) to around 1 ml. When appropriate, proteins were biotinylated with homemade BirA ligase, 100 μM biotin, 40 mM bicine pH 8.3, 10 mM ATP, and 10 mM magnesium acetate at 4 °C overnight. All proteins were further purified by size-exclusion chromatography (SEC) using Superdex Increase S200 or S75, as appropriate (GE Healthcare). All proteins were kept at 4 °C for up to two weeks or as aliquots at −80 °C for more extended storage.

### Surface plasmon resonance

A GE Biacore T100 was used to measure the K_D_ using equilibrium methods. Approximately 200 resonance units (RU) of human CD45 ECD, E3/49K was immobilized on an SA-chip (GE Healthcare), including a reference channel with an unrelated protein. SPR runs were performed in HBS-P+ (GE Healthcare). All measurements were made with twofold serial dilutions using 30 s association at 30 μl/min flow rate, followed by a dissociation time of more than 120 s at 30 μl/min and 25 °C. Regeneration was performed using 0.1 M glycine pH 3 for 30 s at 30 μl/min. Measurement of titrations at equilibrium was used to determine the K_D_ using Biacore Evaluation Software (2.0.4, GE Healthcare). All measurements were repeated twice.

### Cryo-EM sample preparation, data collection, processing, and 3D reconstruction

To prepare the cryo-EM specimens, the complex sample was supplemented to a final concentration of 0.01% FOM immediately before freezing. 3.0 μL of the sample was applied to a glow-discharged 200 mesh holey carbon grid (Quantifoil R 1.2/1.3), and the excess sample was blotted to a filter paper for 0.5 sec and 20–25 force before plunge freezing using Vitrobot Mark IV (Thermo) at 4 °C and 100% humidity. Grids were screened on cEMc’s Glacios 200kV equipped with Gatan K3 camera, and cryo-EM movies were collected using Janelia’s Titan Krios2 operated at 300 kV and equipped with Gatan K3 camera in counting mode and Gatan BioQuantum energy filter with a slit width of 20 eV. Magnification was set to 105,000 × and a super-resolution pixel size of 0.4155-Å. Movies were recorded using serialEM (4.0) at 50 frames at 1.2 e/Å^2^ dose per frame (total dose 60 e/Å^2^) and a defocus range between 0.8 and 2.0 μm. A beam image shift was used with an active calibration to collect three shots per hole three by three holes per stage shift and autofocus.

Collected movies were processed and assessed with cryoSPARC Live (4.3.1) (40). Patch motion correction and patch contrast transfer function (CTF) parameters were estimated with a default setting. A curated 15,166 movies from a 0-degree collection and 13,482 curated movies from a 35-degree collection were used for the downstream processing with cryoSPARC. Particles were picked using Topaz particle detection trained on particles cleaned via 2D sorting from preliminary experiments and extracted with a box size of 416 pixels, binned to 208 pixels (41). Multiple rounds of 2D classifications were used to remove extracted buffer features and contaminants, yielding 182,717 particles from the 0-degree collection (group 1) and 238,660 (groups 2 and 3) particles from the tilted collection, including 99,150 “rare” views (group 2). After rebalancing non-rare views (groups 1 and 3) and splitting into 5,000 particle sets, an ab initio volume was reconstructed by mixing “rare” group 2 with different groups 1 and 3 ratios until nonuniform refinement showed the most complete angular distribution. At this stage, we had 229,150 particles at ~ 4.1-Å, which we further refined with non-uniform and local refinements, followed by deepEMhancer refinement, which yielded a 3.9-Å. This map was then used for hetero refinement in 3D against “junk” particles, which yielded 386,781 particles. The final map was generated with rounds of additional non-uniform and local refinement with a box size of 416 pixels, binned to 288 pixels, which yielded a 3.8-Å nominal resolution map constructed from 377,081 final particles, as determined by gold-standard Fourier shell correlation (FSC) using the 0.143 criteria. The final map was sharpened with the half-map-based2 deepEMhancer (42) for model building and overall map representation, and the half-map-based Phenix v1.21.1 (43) local anisotropic sharpening for model building, visualization of local maps overlaid on the structural model and real-space refinement.

### Model building and refinement

The crystal structures of the human CD45 (PDB ID: 5FMV) and AlphaFold2 (19) model of E3/49K were split into individual domains and docked into the 3D reconstructions using UCSF ChimeraX (44) and deepEMhancer (42) sharpened map for an initial interpretation of the map. The overall structure was fitted into the cryo-EM map with segmental rigid-body fits in Coot (45). The model was refined iteratively using Coot for manual building and Phenix for real-space refinement (40). The structure figures were made using UCSF ChimeraX (44). The buried surface area and contact mapping were calculated using Contacts in CCP4i (8.0.015) with a < 3.7-Å contact cut-off and PDBePISA (www.ebi.ac.uk/pdbe/pisa) (46). Non-polar (dSASA_hphobic) and polar (dSASA_polar) surface areas and other interface parameters were calculated using Rosetta Interface Analyzer (47).

### Statistics

Unless otherwise noted, all figures represent at least three (in vitro) biological replicate experiments. Statistical analysis was assayed by one-way ANOVA, multiple comparisons were conducted using Fisher’s least significant difference (LSD) test using GraphPad Prism (9.0). In all figures *P < 0.05; **P < 0.01; ***P < 0.001; ****P < 0.0001. Data are represented as mean ± s.d., unless otherwise stated.

## Supplementary Material

2

3

4

## Data Availability

The cryo-EM maps have been deposited in the Electron Microscopy Data Bank (EMDB) under accession code EMD-43497. The model coordinates have been deposited in the Protein Data Bank (PDB) under accession code 8VSE. Other data and materials are available in Data file S1 or from the corresponding author upon reasonable request.
