# Supplementary material for: Orientation dependent CD45 inhibition with viral and engineered ligands
Source: Sci Immunol. Author manuscript; Available in PMC 2024 Nov 5. (PMC11537708; doi:10.1126/sciimmunol.adp0707)
Supplement: 2 [file NIHMS2030954-supplement-2.pdf]

Materials Design Analysis Reporting (MDAR) Checklist for Authors

The MDAR framework establishes a minimum set of requirements in transparent reporting applicable to studies in the life sciences (see Statement of Task: doi:10.31222/osf.io/9sm4x.). The MDAR checklist is a tool for authors, editors and others seeking to adopt the MDAR framework for transparent reporting in manuscripts and other outputs. Please refer to the MDAR Elaboration Document for additional context for the MDAR framework.

Materials

Antibodies	Yes (indicate where provided: section/paragraph)	n/a
For commercial reagents, provide supplier name, catalogue number and RRID, if available.	Yes (Supplementary Table S4 and Materials and Methods)	

Cell materials	Yes (indicate where provided: section/paragraph)	n/a
**Cell lines:** Provide species information, strain. Provide accession number in repository **OR** supplier name, catalog number, clone number, **OR** RRID	Yes (Supplementary Table S4 and Materials and Methods)	
**Primary cultures:** Provide species, strain, sex of origin, genetic modification status.	Yes (Supplementary Table S4 and Materials and Methods)	

Experimental animals	Yes (indicate where provided: section/paragraph)	n/a
**Laboratory animals:** Provide species, strain, sex, age, genetic modification status. Provide accession number in repository **OR** supplier name, catalog number, clone number, **OR** RRID		N/A
**Animal observed in or captured from the field:** Provide species, sex and age where possible		N/A
**Model organisms:** Provide Accession number in repository (where relevant) **OR** RRID		N/A

Plants and microbes	Yes (indicate where provided: section/paragraph)	n/a
**Plants:** provide species and strain, unique accession number if available, and source (including location for collected wild specimens)		N/A
**Microbes:** provide species and strain, unique accession number if available, and source		N/A

Human research participants	Yes (indicate where provided: section/paragraph)	n/a
Identify authority granting ethics approval (IRB or equivalent committee(s), provide reference number for approval.		N/A
Provide statement confirming informed consent obtained from study participants.		N/A
Report on age and sex for all study participants.		N/A

Design

Study protocol	Yes (indicate where provided: section/paragraph)	n/a
For clinical trials, provide the trial registration number **OR** cite DOI in manuscript.		N/A

Laboratory protocol	Yes (indicate where provided: section/paragraph)	n/a
Provide DOI or other citation details if detailed step-by-step protocols are available.		N/A

Experimental study design (statistics details)	Yes (indicate where provided: section/paragraph)	n/a
State whether and how the following have been done**, or** if they were not carried out.	Yes (Materials and Methods/ Statistics)	
Sample size determination		N/A
Randomisation		N/A
Blinding		N/A
Inclusion/exclusion criteria		N/A

Sample definition and in-laboratory replication	Yes (indicate where provided: section/paragraph)	n/a
State number of times the experiment was replicated in laboratory	Yes (Figure legends)	
Define whether data describe technical or biological replicates	Yes (Figure legends)	

Ethics	Yes (indicate where provided: section/paragraph)	n/a
Studies involving human participants: State details of authority granting ethics approval (IRB or equivalent committee(s), provide reference number for approval.		N/A
Studies involving experimental animals: State details of authority granting ethics approval (IRB or equivalent committee(s), provide reference number for approval.		N/A
Studies involving specimen and field samples: State if relevant permits obtained, provide details of authority approving study; if none were required, explain why.		N/A

Dual Use Research of Concern (DURC)	Yes (indicate where provided: section/paragraph)	n/a
If study is subject to dual use research of concern, state the authority granting approval and reference number for the regulatory approval		N/A

Analysis

Attrition	Yes (indicate where provided: section/paragraph)	n/a
State if sample or data point from the analysis is excluded, and whether the criteria for exclusion were determined and specified in advance.		N/A

Statistics	Yes (indicate where provided: section/paragraph)	n/a
Describe statistical tests used and justify choice of tests.	Yes (Materials and Methods/ Statistics)	

Data Availability	Yes (indicate where provided: section/paragraph)	n/a
State whether newly created datasets are available, including protocols for access or restriction on access.	Yes (raw files are included in Supplementary Data File S1 and cryo-EM structure is available under PDB ID: 8VSE)	
If data are publicly available, provide accession number in repository or DOI or URL.		N/A
If publicly available data are reused, provide accession number in repository or DOI or URL, where possible.		N/A

Code Availability	Yes (indicate where provided: section/paragraph)	n/a
For all newly generated code and software essential for replicating the main findings of the study:		N/A
State whether the code or software is available.		N/A
If code is publicly available, provide accession number in repository, or DOI or URL.		N/A

Reporting

Adherence to community standards	Yes (indicate where provided: section/paragraph)	n/a
MDAR framework recommends adoption of discipline-specific guidelines, established and endorsed through community initiatives. Journals have their own policy about requiring specific guidelines and recommendations to complement MDAR.		N/A
State if relevant guidelines (eg., ICMJE, MIBBI, ARRIVE) have been followed, and whether a checklist (eg., CONSORT, PRISMA, ARRIVE) is provided with the manuscript.		N/A

Article information:
