# Supplementary material for: Orientation dependent CD45 inhibition with viral and engineered ligands
Source: Sci Immunol. Author manuscript; Available in PMC 2024 Nov 5. (PMC11537708; doi:10.1126/sciimmunol.adp0707)

Supplementary Materials for

**Orientation dependent CD45 inhibition by viral and engineered ligands**

Marta T. Borowska and Liu D. Liu *et al.*

Corresponding author: K. Christopher Garcia, [kcgarcia@stanford.edu](mailto:kcgarcia@stanford.edu)

**This PDF file includes:**

Figures S1 to S7

Tables S1 to S5

**Figure S1**

**
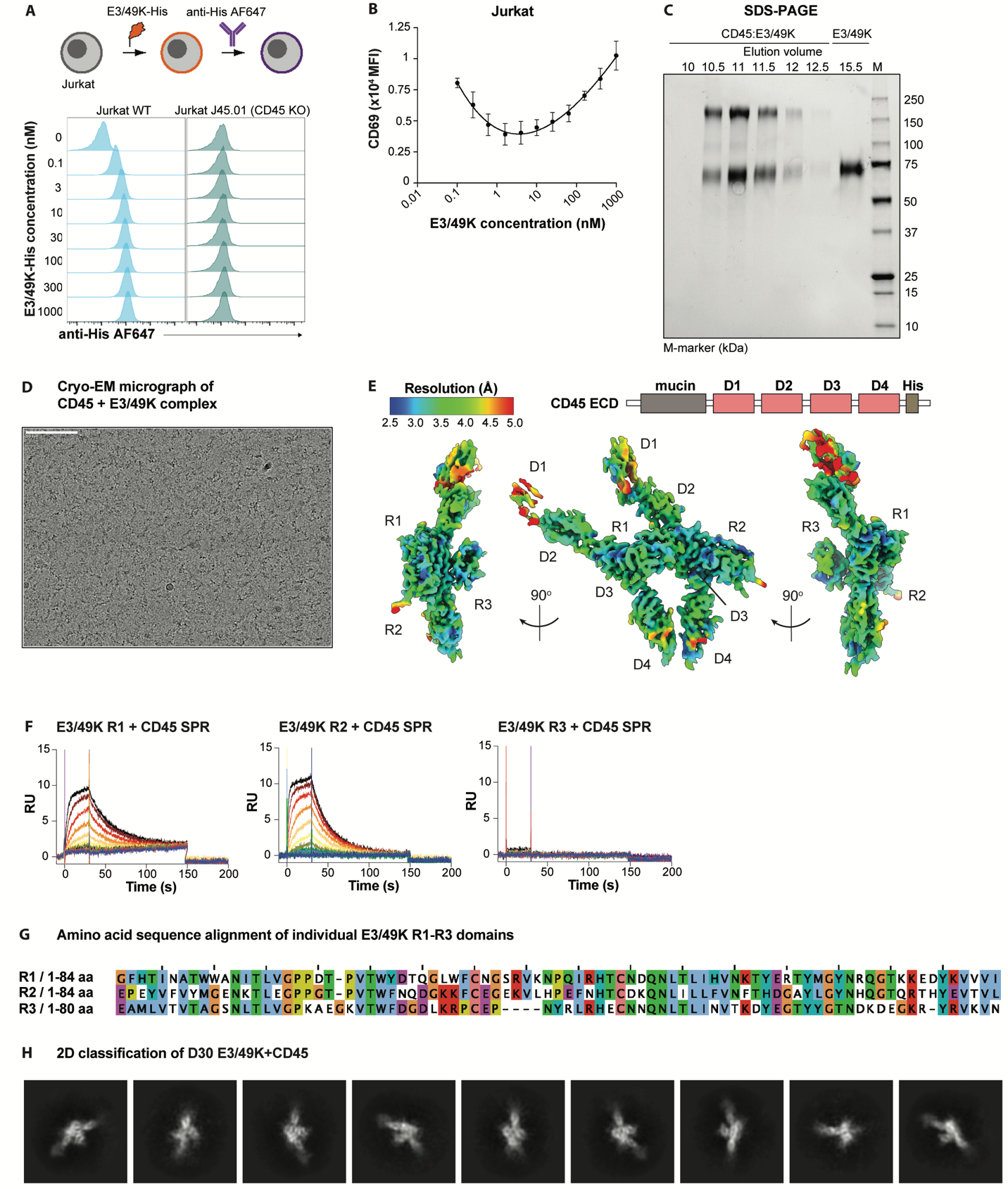
**

**Figure S1. Biochemistry and cryo-EM imaging of purified CD45:E3/49K complex. (A)** Titration of surface staining with E3/49K of Jurkat WT and Jurkat CD45KO T cells. **(B)** Dose dependence of E3/49K on CD69 expression upon OKT3 stimulation in Jurkat T cells. **(C)** SDS-PAGE purification of the CD45:E3/49K complex from Superdex 200 gel filtration. **(D)** Representative micrograph from cryo-EM data collection. **(E)** Local resolution estimates by cryoSPARC on the surface representation of the deepEMhancer sharpened map. The blue-green-red gradient corresponds to 2.5 to 5-Å resolution, respectively. (*top right*) Cartoon representation of the CD45 construct used for cryo-EM structure. **(F)** SPR plots of CD45 and individual domains of E3/49K: R1 (*left*), R2 (*middle*), and R3 (*right*). **(G)** Amino acid sequence alignment of E3/49K domains R1-R3 using Jalview (2.11.0). **(H)** 2D classification of E3/49K ECD strain D30 + CD45 ECD complex.

**Figure S2**

**
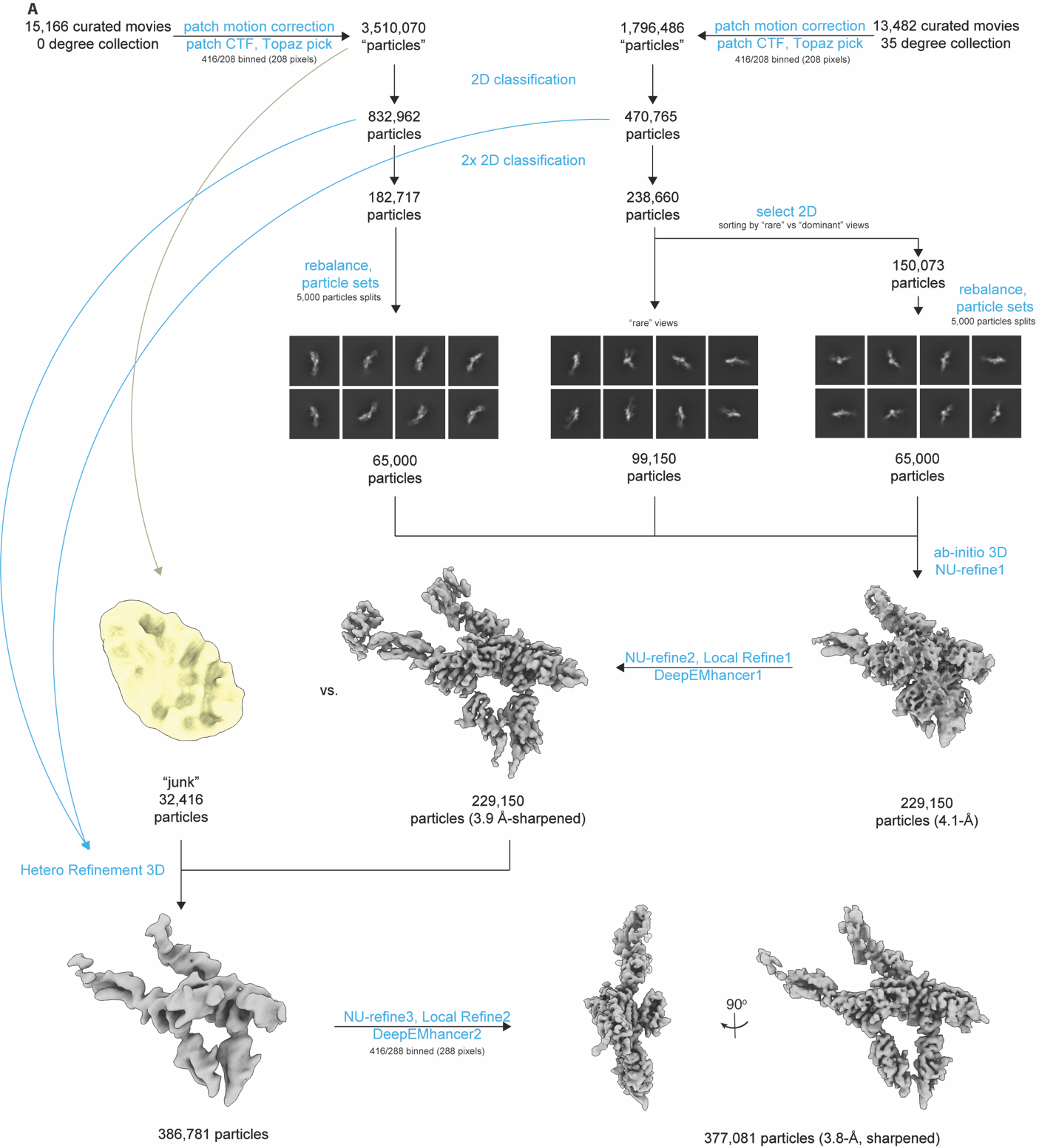
**

**Figure S2. Cryo-EM data processing scheme. (A)** Cryo-EM data processing strategy shows the workflow with representative 2D classifications and 3D reconstructions during the data processing.

**Figure S3**

**
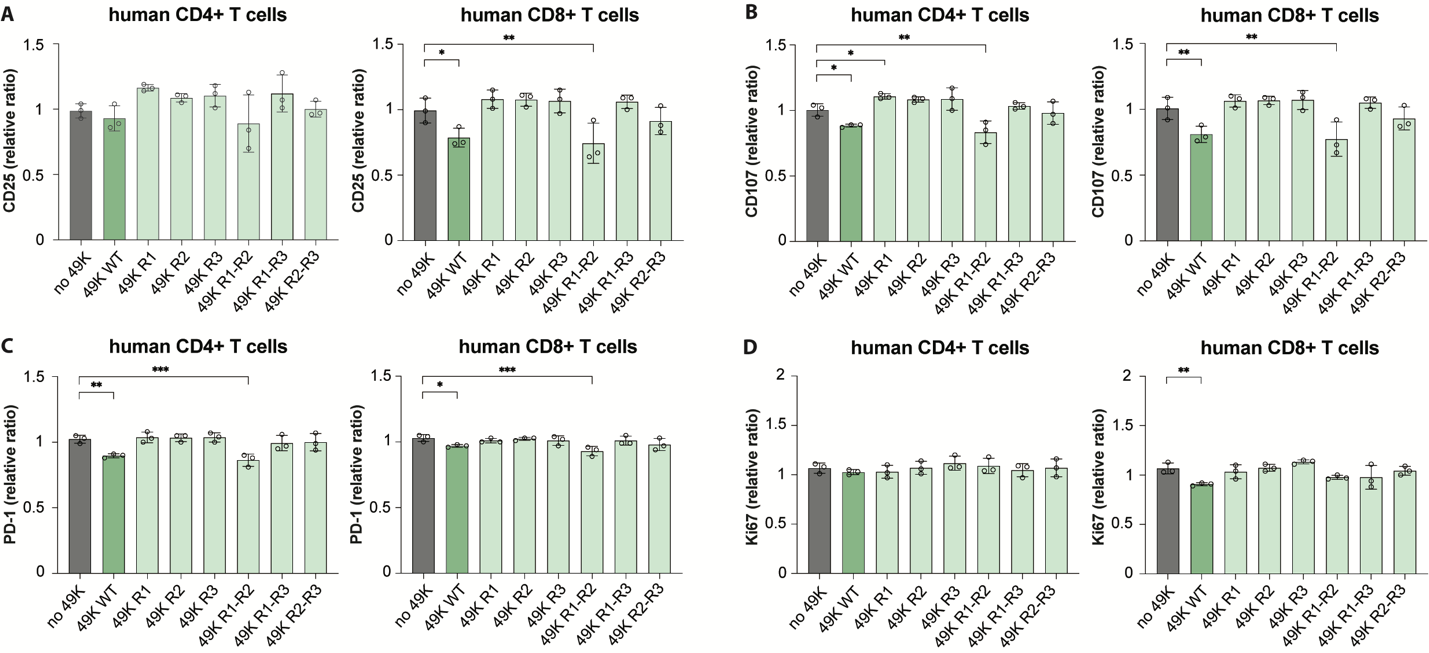
**

**Figure S3. E3/49K domain truncations effect on T cells. (A-D)** Different markers expression upon pretreatment with different E3/49K domain truncations then stimulated with OKT3 in CD4+ T cells (*left*) or in CD8+ T cells (*right*) from PBMCs. **(A)** CD25, **(B)** CD107, **(C)** PD-1, and **(D)** Ki67 markers. Data are mean ± s.d. from n = 3 different donors. Statistical significance is determined by one-way ANOVA with Fisher’s LSD multiple comparison test (ns > 0.05; *P < 0.05; **P < 0.01, ***P < 0.001; ****P < 0.0001).

**Figure S4
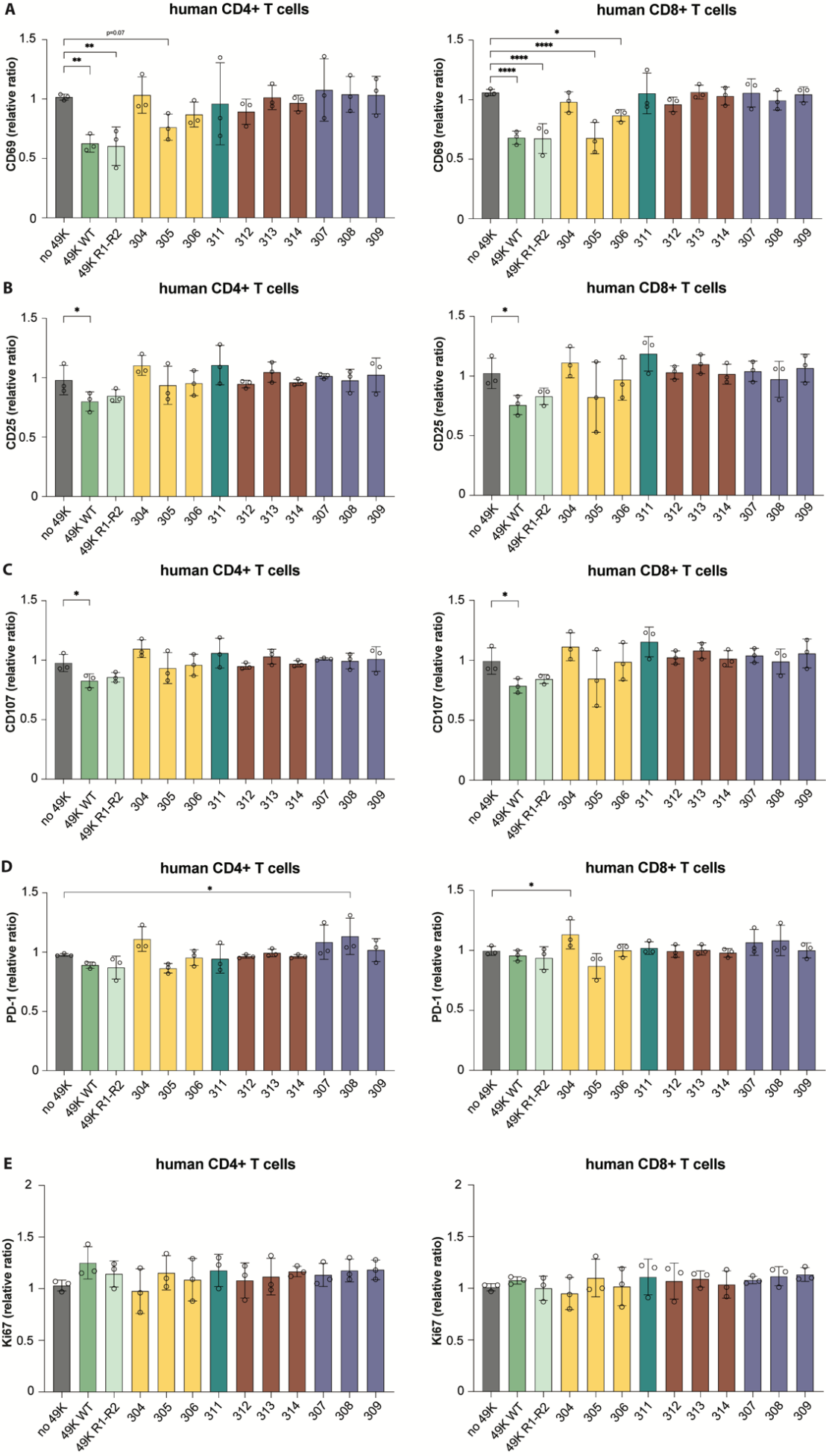
**

**Figure S4. E3/49K surrogates and its VHH mimics impair CD45 phosphatase activity in PBMCs. (A-E)** Different markers expression upon pretreatment with different VHH mimics then stimulated with OKT3 in CD4+ T cells (*left*) or in CD8+ T cells (*right*) from PBMCs. **(A)** CD69, **(B)** CD25, **(C)** CD107, **(D)** PD-1, and **(E)** Ki67 markers. Data are mean ± s.d. from n = 3 different donors. Statistical significance is determined by one-way ANOVA with Fisher’s LSD multiple comparison test (ns > 0.05; *P < 0.05; **P < 0.01, ***P < 0.001; ****P < 0.0001).

**Figure S5**

**
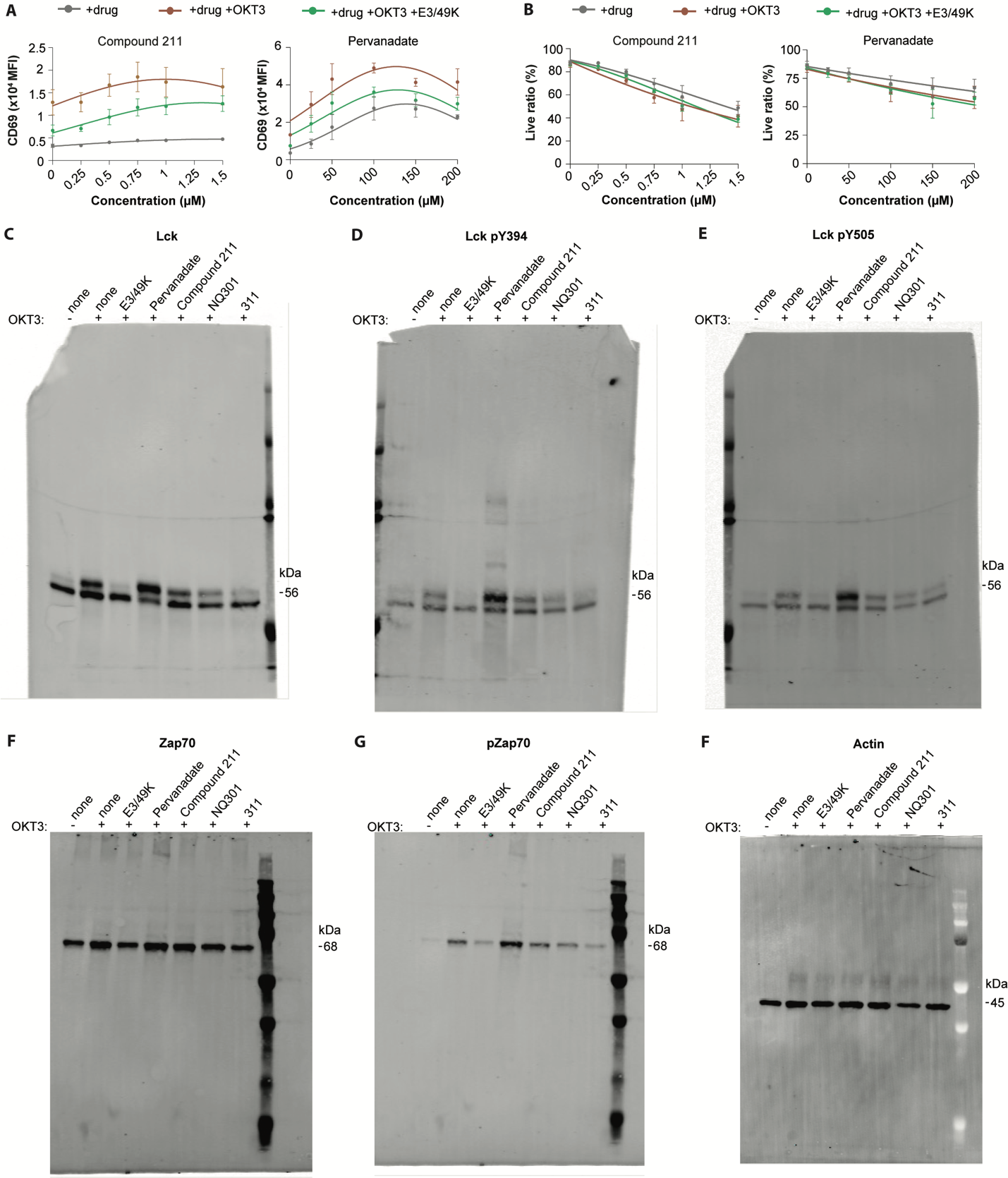
**

**Figure S5. Dose optimization of common phosphatase inhibitors. (A)** CD69 expression in Jurkat T cells upon treatment with different doses of phosphatase inhibitors: Compound 211 (*left*), Pervanadate (*right*). **(B)** Toxicity measure in Jurkat T cells upon treatment with different doses of phosphatase inhibitors: Compound 211 (*left*), Pervanadate (*right*). **(C-F)** Raw protein immunoblot of **(C)** total Lck, **(D)** Lck pY394, **(E)** Lck pY505, **(F)** total Zap70, **(G)** and phosphorylated Zap70, and **(F)** actin as loading control in Jurkat cells, also shown edited in Fig. 4A.

**Figure S6**

**
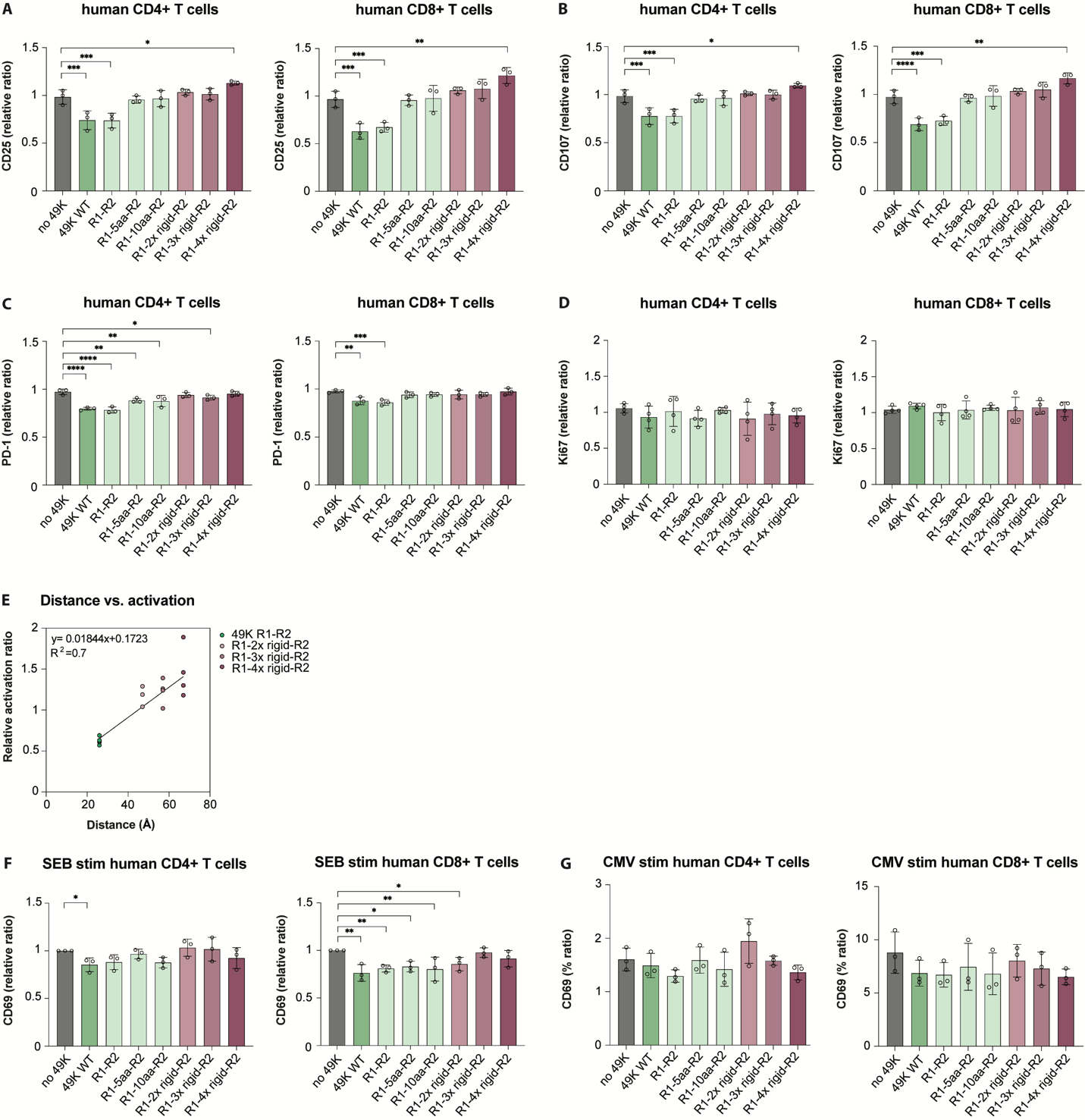
**

**Figure S6. CD45 distancing tunes signaling in Jurkat T cells. (A-D)** Different markers expression upon pretreatement with E3/49K with different types of linkers then stimulated with OKT3 and assayed for different markers in CD4+ T cells (*left*) or in CD8+ T cells (*right*) from PBMCs. **(A)** CD25, **(B)** CD107, **(C)** PD-1, and **(D)** Ki67 markers. Data are mean ± s.d. from n = 3 different donors. Statistical significance is determined by one-way ANOVA with Fisher’s LSD multiple comparison test (ns > 0.05; *P < 0.05; **P < 0.01, ***P < 0.001; ****P < 0.0001). **(E)** Distance vs activation analysis of CD69 activation levels of CD8+ T cells and the approximate distance between R1 and R2 domains with different rigid linkers compared to wild type. Data fitted with linear fitting. **(F-G)** CD69 expression upon pretreatment with E3/49K followed by **(F)** SEB or **(G)** CMV stimulation in CD4+ T cells (*left*) or in CD8+ T cells (*right*) from PBMCs. Data are mean ± s.d. from n = 3 different donors. Statistical significance is determined by one-way ANOVA with Fisher’s LSD multiple comparison test (ns > 0.05; *P < 0.05; **P < 0.01, ***P < 0.001; ****P < 0.0001).

**Figure S7**


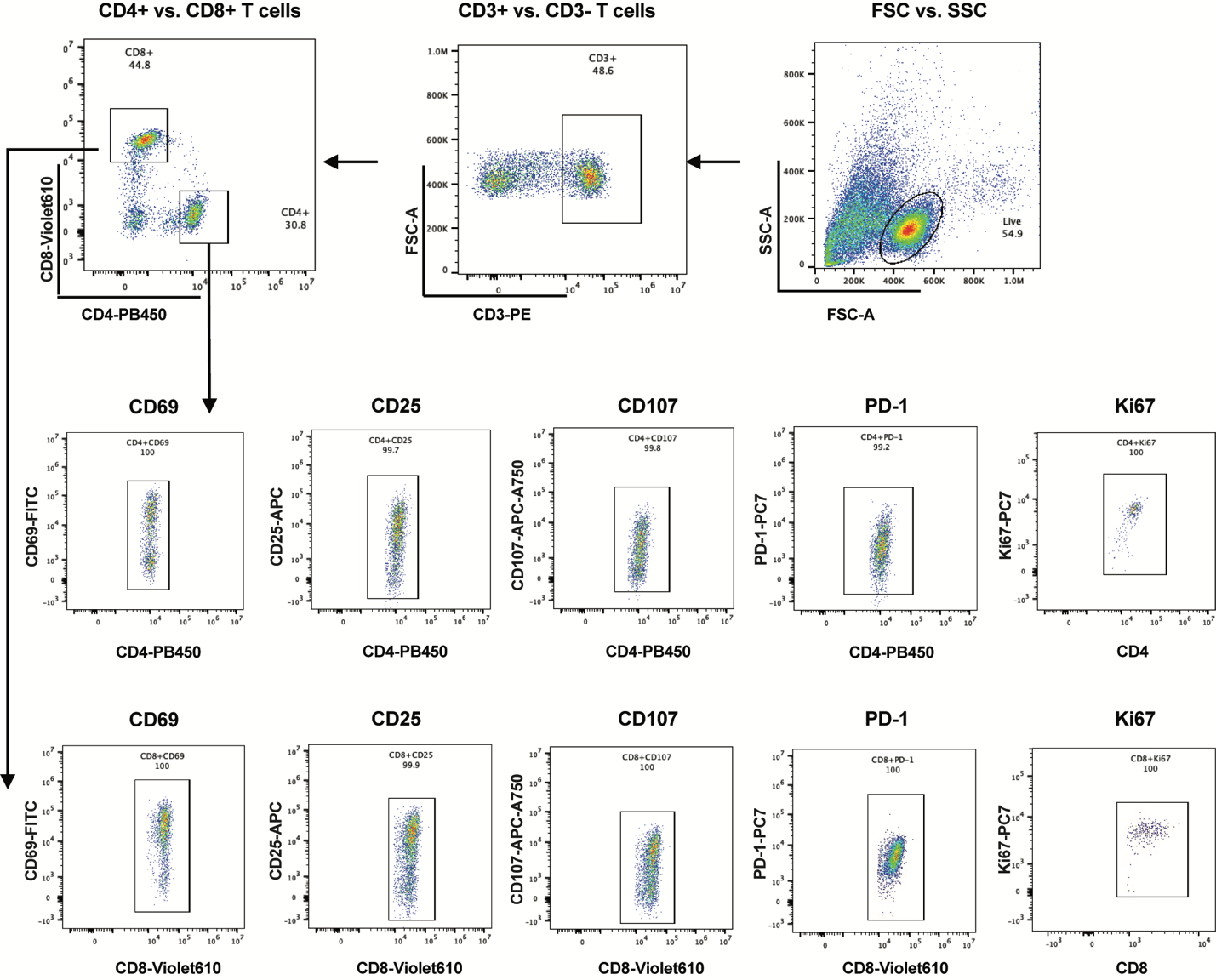


**Figure S7. Representative gating strategy for expression of markers in the CD4+ T cells and CD8+ T populations in PBMCs.**

**Table S1. Cryo-EM data collection and refinement statistics.**

**
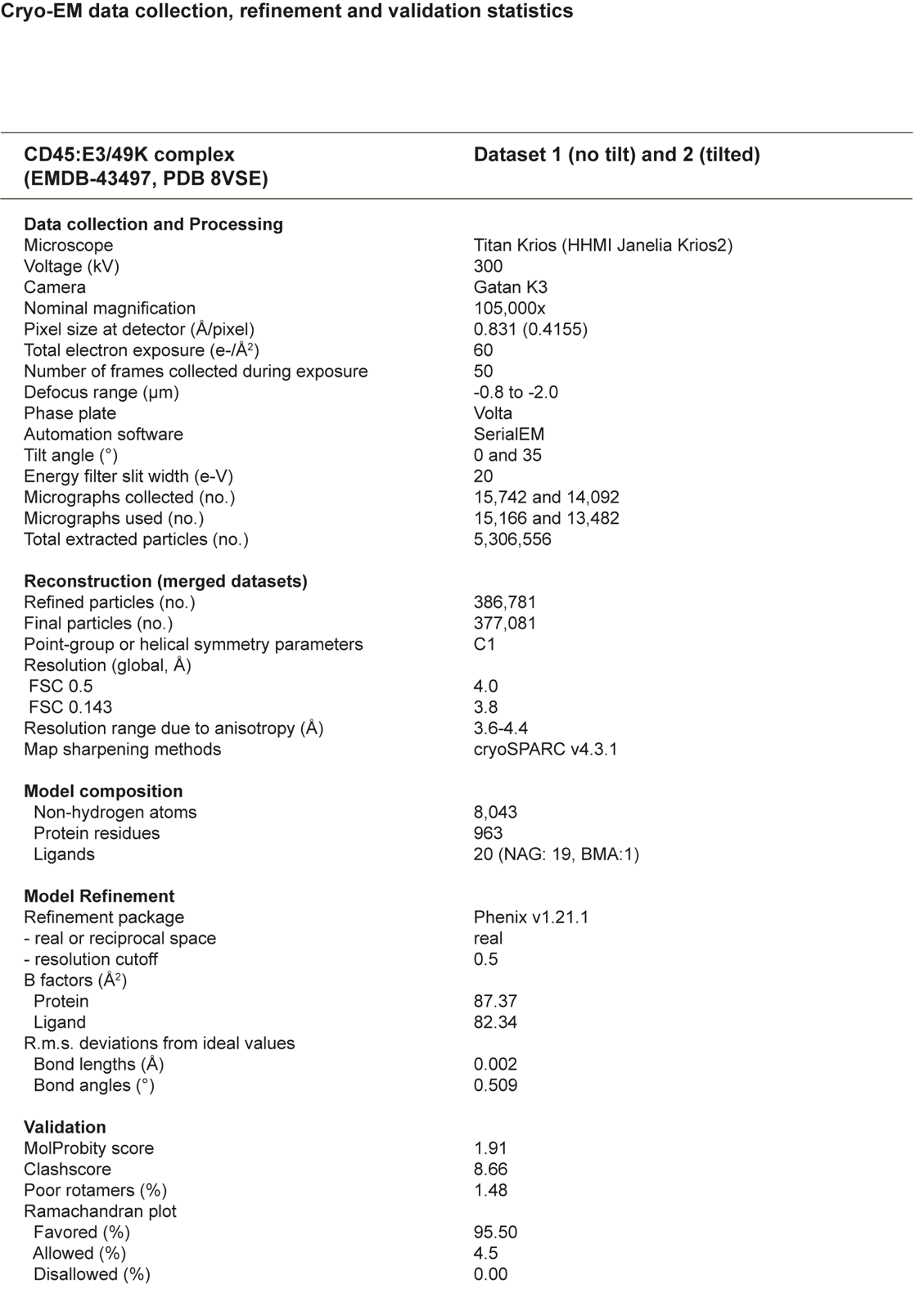
**

**Table S2. Rosetta Interface Analyzer statistics: including buried surface areas and shape complementarity at the CD45:E3/49K intermolecular interface.**

**
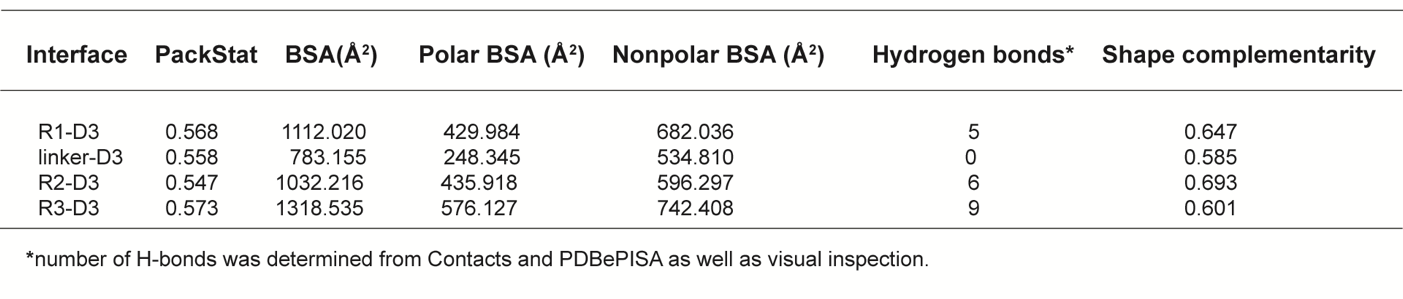
**

**Table S3. Contact atoms and distances of the CD45:E3/49K intermolecular interface calculated by Contacts in CCP4i (8.0.015) with a < 3.7-Å contact cut-off. *** indicates H-bond, and */** indicates a probability of H-bonds.**


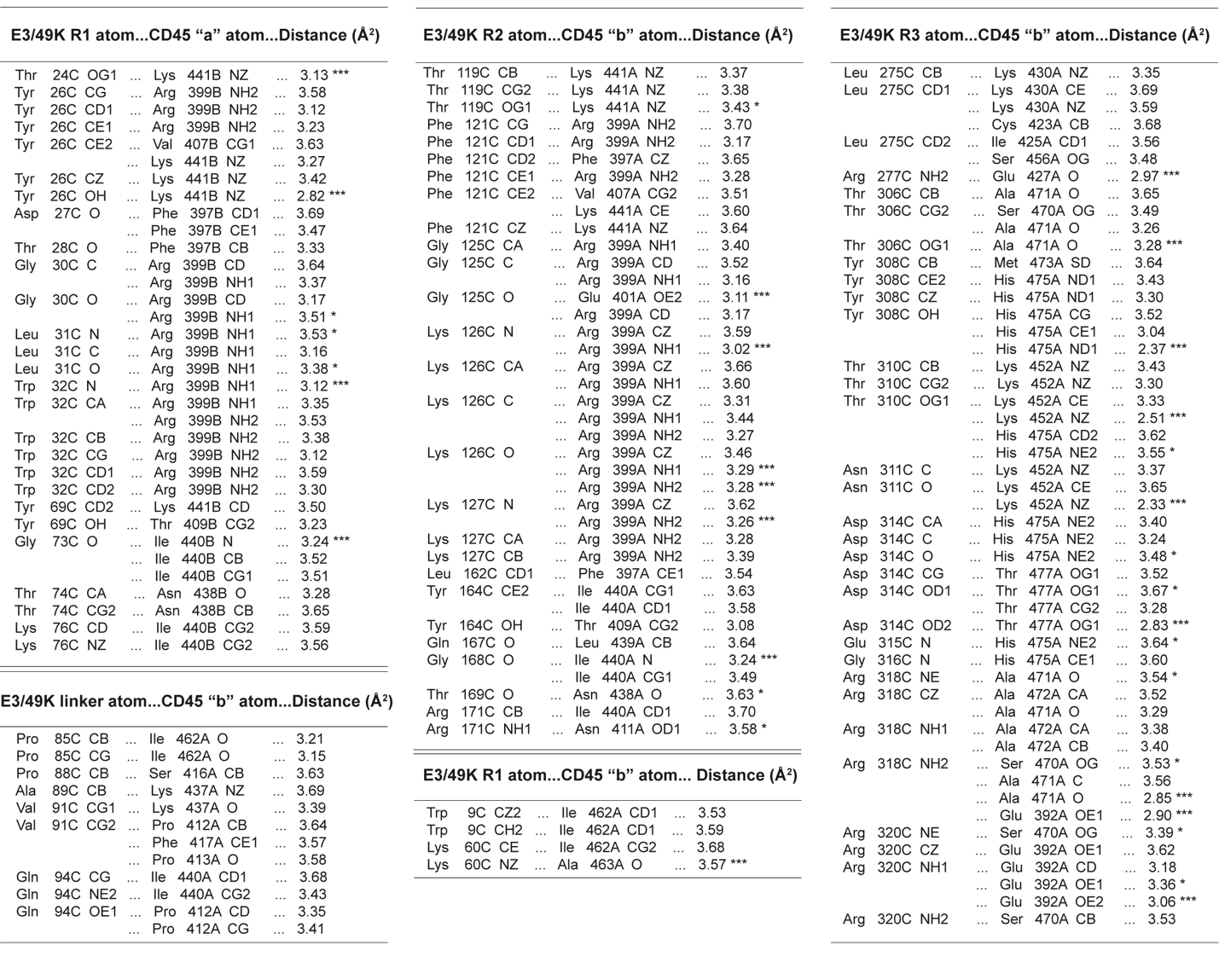


**Table S4. Antibodies, cells are reagents for cells used in the study.**


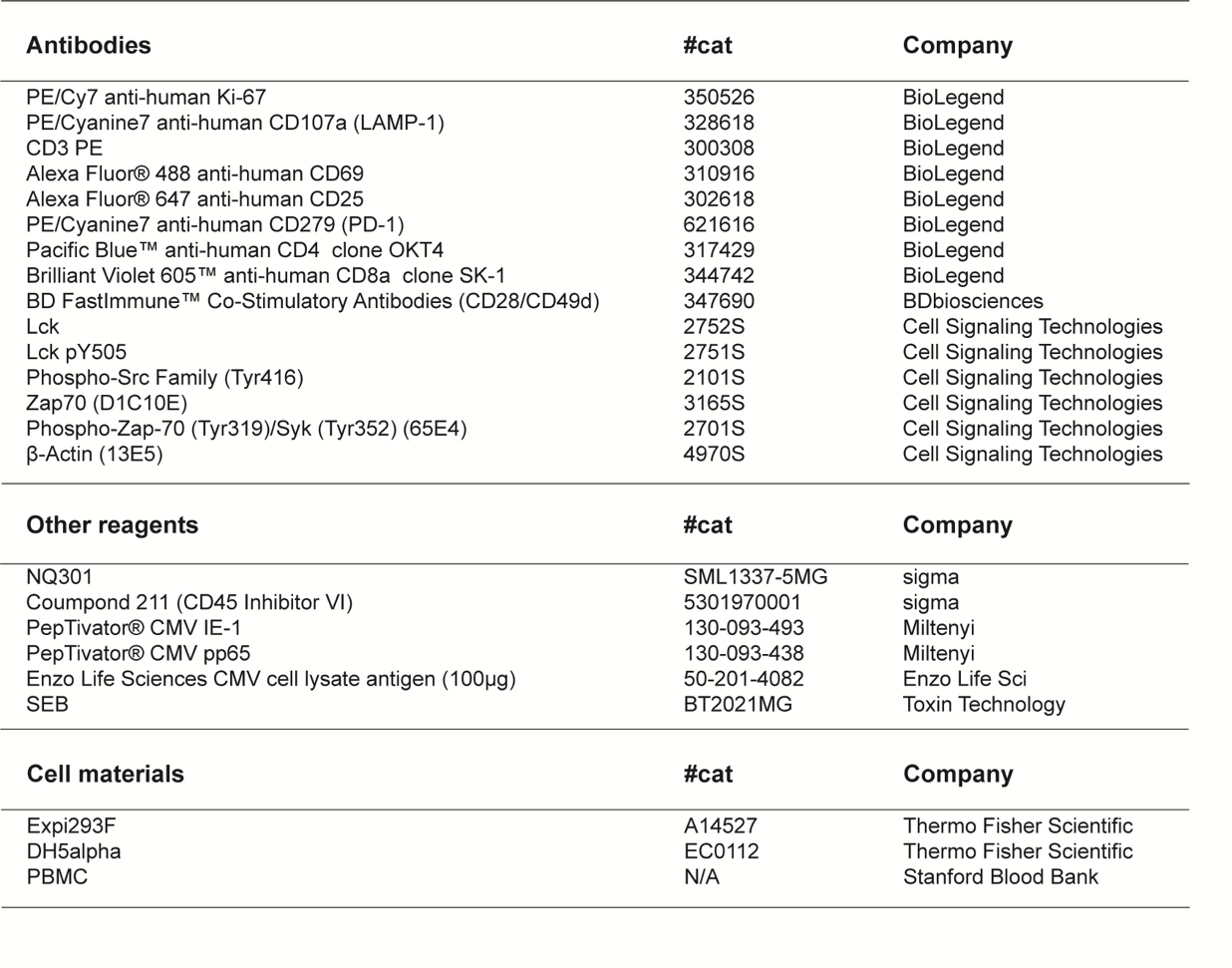


**Table S5. Amino acid sequences of constructs used in the study.**


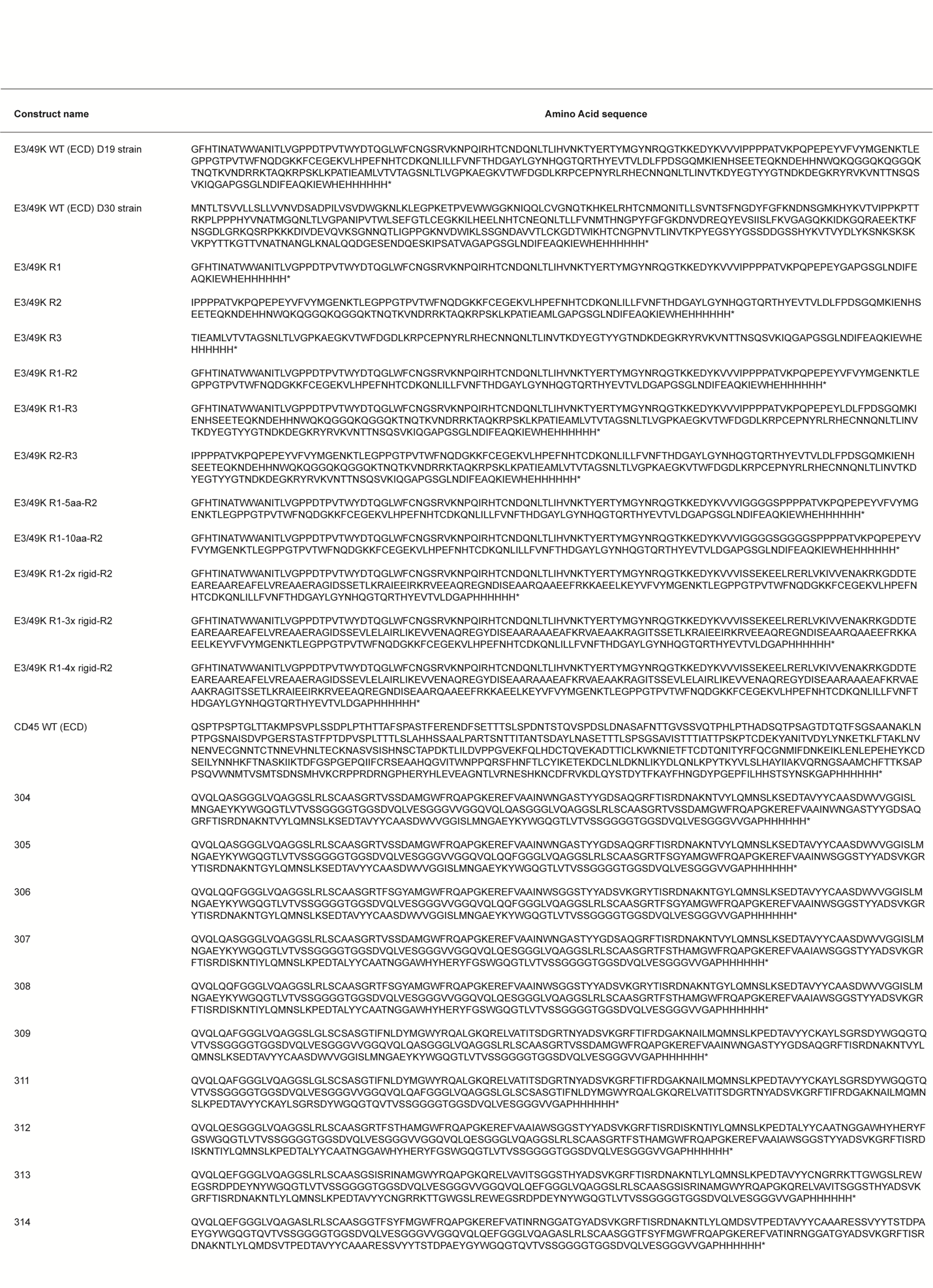

Supplement: 4 [file NIHMS2030954-supplement-4.docx]
